# Supplementary material for: The NP protein of Newcastle disease virus dictates its oncolytic activity by regulating viral mRNA translation efficiency
Source: PLoS Pathog. 2024 Feb 20;20(2):e1012027. doi: 10.1371/journal.ppat.1012027 (PMC10906838; doi:10.1371/journal.ppat.1012027)
Supplement: S1 Table — (DOCX) [file ppat.1012027.s001.docx]

**S1 Table. Construction strategy and primers for generating recombinant viruses**

| Application | Primer | Sequence (5’-3’) |
| --- | --- | --- |
| 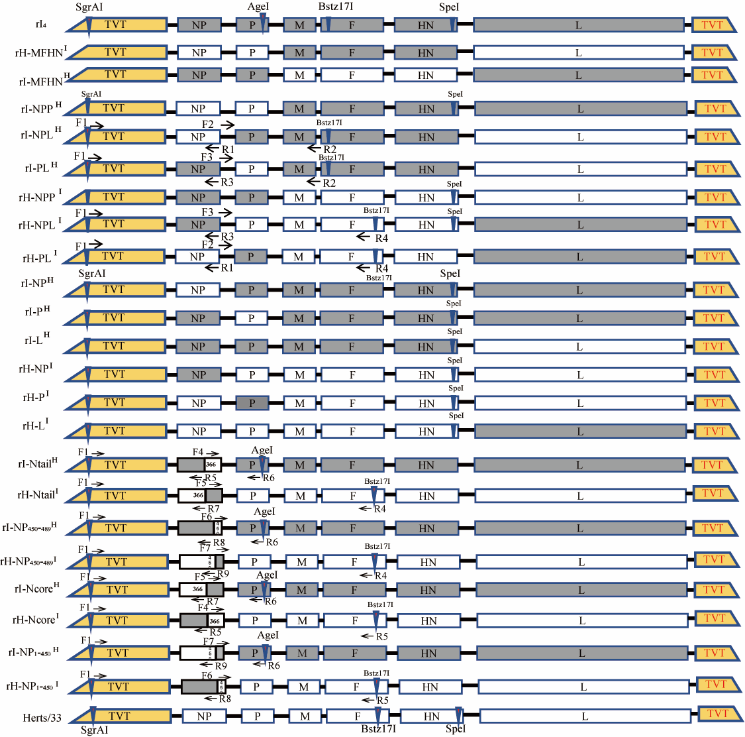  Recombinant viruses were generated by exchanging a single NP, P, M, F, HN, or L gene or a combination thereof between I_4_ and Herts/33. Restriction enzymes including SgrAⅠ (15735), AgeⅠ (2879), Bstz17Ⅰ (4705), ApaⅠ (2289), PacⅠ (2899), and SpeⅠ (8094) were used. Plasmid rI-NPP^H^ was created by cleaving rH-MFHN^I^ and rI_4_ with SgrAI and SpeI and exchanging the L gene. Notably, the sequence of I_4_ and Herts/33 HN gene are identical at 28 nucleotides after SpeⅠ. Fragments 1 and 2 were obtained by PCR using F1 and R1 with rI_4_ as a template and F2 and R2 with rHerts/33 as a template, respectively. Fragment 1 and fragment 2 were overlapped and homologously recombined with the fragment after enzymatic cleavage of rH-MFHN^I^ by SgrAⅠ and Bstz17Ⅰ to obtain plasmid rI-NPL^H^. Plasmid rI-NPL^H^ was obtained by PCR using F1 and R3 with rHerts/33 as a template to obtain fragment 3, and F3 and R4 were used to obtain fragment 4 using rH-MFHN^I^ as a template. Fragment 3 and fragment 4 were overlapped and homologously recombined with SgrAⅠ and Bstz17Ⅰ digested rH-MFHN^I^ to obtain plasmid rI-PL^H^. rH-NPP^I^ was obtained from SgrAⅠ and SpeⅠ digested rI-MFHN^H^ and rHerts/33, followed by exchanging the L gene. Fragment 3 was obtained by PCR using F1 and R3 with rI_4_ as a template, fragment 5 was obtained by PCR using F3 and R5 with rHerts/33 as a template. Fragment 3 and fragment 5 were overlapped and homologously recombined with the fragment after enzymatic cleavage of rH-MFHN^I^ by SgrAⅠ and Bstz17Ⅰ to obtain plasmid rH-NPL^I^. Fragment 1 was obtained using F1 and R1 with rI_4_ as a template, fragment 4 was obtained using F2 and R4 with rI-MFHN^H^ as a template. Fragment 1 and fragment 4 were overlapped and homologously recombined with the fragment after enzymatic cleavage of rI-MFHN^H^ by SgrAⅠ and Bstz17Ⅰ to obtain plasmid rH-PL^I^.  All of the following recombinant viruses were obtained by enzymatic cleavage of the corresponding backbone viruses by SgrAⅠ and SpeⅠ and exchange of L genes. For example, rI-NP^H^ was derived from rI-NPL^H^ and rI_4_, rI-P^H^ from rI-PL^H^ and rI_4_, rI-L^H^ from rHerts/33 and rI_4_, rH-NP^I^ from rH-NPL^I^ and rHerts/33, rH-P^I^ from rH-PL^I^ and rHerts/33, and rH-L^I^ from rHerts/33 and rI_4_.  The construction strategy for generating recombinant viruses by exchanging different domains of the NP protein between rI_4_ and rHerts/33 strains is as follows. Fragment 5 was obtained using F1 and R5 primers with rI_4_ as a templates, while fragment 6 was obtained using F4 and R6 primers with rI-NP^H^ as template. Fragments 5 and 6 were then overlapped and homologously recombined with the SgrAI and AgeI digested rI_4_ to obtain rI-Ntail^H^. Similarly, fragment 7 was obtained using F1 and R7 primers with rHerts/33 as a template, and fragment 8 was obtained using F5 and R4 primers with rH-NP^I^ as a template. Fragments 7 and 8 were overlapped and recombined homologously with the SgrAⅠ and Bstz17Ⅰ digested rHerts/33 to obtain rH-Ntail^I^. Fragment 9 was obtained using F1 and R8 primers with rI_4_ as a template, while fragment 10 was obtained using F6 and R6 primers with rI-NP^H^ as template. Fragments 9 and 10 were then overlapped and homologously recombined with the SgrAⅠ and AgeⅠ digested rI_4_ to obtain rI-NP_450-489_^H^. Fragment 11 was obtained using F1 and R9 primers with rHerts/33 as a template, while fragment 12 was obtained using F7 and R4 primers with rI-NP^H^ as a template. Fragments 11 and 12 were then overlapped and homologously recombined with SgrAⅠ and Bstz17Ⅰ digested rHerts/33 to obtain rH-NP_450-489_^I^. Fragment 7 was obtained using F1 and R7 primers with rHerts/33 as a template as before, Fragment 13 was obtained using F5 and R6 primers with rI_4_ as a template, Fragments 7 and 13 were then overlapped and homologously recombined with SgrAⅠ and AgeⅠ digested rI_4_ to obtain rI-Ncore^H^. Fragment 5 was obtained using F1 and R5 primers with rI_4_ as a template as before, while fragment 14 was obtained using F4 and R5 primers with rHerts/33 as a template. Fragments 5 and 14 were then overlapped and homologously recombined with the SgrA I and Bstz17Ⅰ digested rHerts/33 to obtain rH-Ncore^I^. Fragment 11 was obtained using F1 and R9 primers with rHerts/33 as a template as before, while fragment 15 was obtained using F7 and R6 primers with rI_4_ as a template. Fragments 11 and 15 were then overlapped and homologously recombined with SgrAⅠ and AgeⅠ digested rI_4_ to obtain rI-NP_1-450_^H^. Fragment 9 was obtained using F1 and R8 primers with rI_4_ as a template, while fragment 16 was obtained using F6 and R5 primers with rHerts/33 as a template. Fragments 9 and 16 were then overlapped and homologously recombined with the SgrAⅠ and Bstz17Ⅰ digested rHerts/33 to obtain rH-NP_1-450_^I^. | F1 | GGACGCATCGTGGCCGGCAT |
|  | R1 | GCAGGCTGGGTGTAGTCGATCAATACCCCCAGTCGGTGT |
|  | F2 | ACACCGACTGGGGGTATTGATCGACTACACCCAGCCTGCC |
|  | R2 | GACCCTGTCTGAGACGAGGT |
|  | F3 | ACACTGACTGGGGGTACTGACCGACAACACCCAGTCTGCC |
|  | R3 | GGCAGACTGGGTGTTGTCGGTCAGTACCCCCAGTCAGTGT |
|  | F4 | GAGCACATCATTCTGGAGACTTGGAGTAGAGTATGCTCAGGCTCAGGG |
|  | R5 | CCCTGAGCCTGAGCATACTCTACTCCAAGTCTCCAGAATGATGTGCTC |
|  | R6 | TCAATTCAGAAGGGTGCTGC |
|  | F5 | GAGCACATCATTTTGGAGACTTGGAGTAGAGTATGCTCAGGCTCAGGG |
|  | R7 | CCCTGAGCCTGAGCATACTCTACTCCAAGTCTCCAAAATGATGTGCTC |
|  | F6 | GAGACCCAATTCCTGGATTTTATGAGAGCGGTGGCAAATAGCA |
|  | R8 | TATTTGCCACCGCTCTCATAAAATCCAGGAATTGGGTCTCCCC |
|  | F7 | GGAGACCCAATTCCTGGATTTGATGAGAGCAGTGGCGAACAGC |
|  | R9 | CTGTTCGCCACTGCTCTCATCAAATCCAGGAATTGGGTCTCCCC |
| H-NP_C479G_ | F | GCCTCCCTCAACTCCTGGGGCGTCCCAAGGCAA |
|  | R | CCCAGGAGTTGAGGGAGGCTCCGGTTGAGTGG |
| I-NP_P464S_ | F | ATGCGGGAAGCGCCAAATTCTGCACAGAGCACCA |
|  | R | ATTTGGCGCTTCCCGCATGCTGTTCGCCACTGCT |
| I-NP_P475S_ | F | ACCCATCTAGAGCCTCCCTCAACCCCTGGGGCAT |
|  | R | GGGAGGCTCTAGATGGGTGGTGCTCTGTGCAGAA |
| H-NP_S464P_ | F | GCATGCGAGAAACGCCAAACCCCGCACAGAGCA |
|  | R | GTTTGGCGTTTCTCGCATGCTATTTGCCACCG |
| H-NP_L450F_ | F | GACCCAATTCCTGGATTTCATGAGAGCGGTGGCAAA |
|  | R | GAAATCCAGGAATTGGGTCTCCCCATCTCCGGCA |
| I-NP_F450L_ | F | GACCCAATTCCTGGATTTAATGAGAGCAGTGGCGA |
|  | R | TAAATCCAGGAATTGGGTCTCCCCATCCCCAGCAT |
| K-NP_L450F_ | F | AGACCCAATTCCTGGATTTCATGAGAGCAGTGGCG |
|  | R | GAAATCCAGGAATTGGGTCTCCCCATCCCTGGCAT |
| L-NP_R450F_ | F | GAGACCCAATTCCTGGATCTGATGAGAGCGGTAGC |
|  | R | AGATCCAGGAATTGGGTCTCCCCATCCCCGGCTTC |
