# Supplementary material for: The NP protein of Newcastle disease virus dictates its oncolytic activity by regulating viral mRNA translation efficiency
Source: PLoS Pathog. 2024 Feb 20;20(2):e1012027. doi: 10.1371/journal.ppat.1012027 (PMC10906838; doi:10.1371/journal.ppat.1012027)

**S2.Table Construction strategy and primers for generating minigenome system-related plasmids.**

| Application | Primer | Sequence (5’-3’) |
| --- | --- | --- |
| pCI-I_4_-NP | pCI-I_4_-NP -F | CTATAGGCTAGCCTCGAGAATTCATGTCGTCTGTTTTCGACGAATACG |
|  | pCI-I_4_-NP | CTCGAAGCGGCCGCCCGGGTCAGTACCCCCAGTCAGTG |
| pCI-I_4_-P | pCI-I_4_-P-F | ACTATAGGCTAGCCTCGAGAATTCATGGCTACTTTTACAGATGCGGAGAT |
|  | pCI-I_4_-P-R | TCTGCTCGAAGCGGCCGCCCGGGTCAACCATTCAGCGCAAGGCG |
| pCI-I_4_-L | pCI-I_4_-L-F | AGTGTGCTGGAATTCGGCTTGATTACAAGGATGACGACGATAAGATGGCGGGCTCCGGT |
|  | pCI-I_4_-L-R | TGCAGAATTCGGCTTTTAAGAGTCATTATTACTGTAATATCCCTTGGCAG |
| pCI-H-NP | pCI-H-NP-F | TAGGCTAGCCTCGAGAATTCATGTCTTCCGTATTCGACGAATACGAG |
|  | pCI-H-NP-R | TCTGCTCGAAGCGGCCGCCCGGGTCAATGATGATGATGATGGTGATACCCCCAGTCGGTGTCAT |
| pCI-H-P | pCI-H-P-F | CACTATAGGCTAGCCTCGAGAATTCATGGCCACCTTTACAGATGCG |
|  | pCI-H-P-R | TCTGCTCGAAGCGGCCGCCCGGGTCAGCCATTCAGCGCAAGGC |
| pCI-H-L | pCI-H-L-F | CACTATAGGCTAGCCTCGAGAATTCGAGCAGAAACTCATCTCTGAAGAGGATCTGATGGCAAGCTCCGGTCCC |
|  | pCI-H-L-R | CTGCTCGAAGCGGCCGCCCGGGTTAAGAGTTATCGTTACTGTAATATCCCTTG |
| TVT-I_4_-GFP/TVT-Herts/33-GFP | TVT-R | TATAGTGAGTCGTATTAAT |
|  | I4-1-F | ATTAATACGACTCACTATAACCAAACAGAGATTTGGTGAATGACAT |
|  | GFP-F | CTACTTGTACAGCTCGTCC |
|  | I4-2-R | GGACGAGCTGTACAAGTAGAGGCAATCGTACGCCAATCAG |
|  | I4-3-F | CTCGCCCTTGCTCACCATATTGGTAGAAGGTTCCCTCAGG |
|  | GFP-R | ATGGTGAGCAAGGGCGAGG |
|  | I4-3-R | GAGATGCCATGCCGACCCACCAAACAGAGAATCTGTGA |
|  | H-1-F | ATTAATACGACTCACTATAACCAAACAAAGATTTGGTGAATGACAGG |
|  | H-1-R | TGGACGAGCTGTACAAGTAGAGGCAATCACATATTAATATGCTTTCC |
|  | H-2-F | TCGCCCTTGCTCACCATGTCAGCAGAAGGCTCTCT |
|  | H-2-R | TGGAGATGCCATGCCGACCCACCAAACAGAGATTCTGTGAGGTACG |


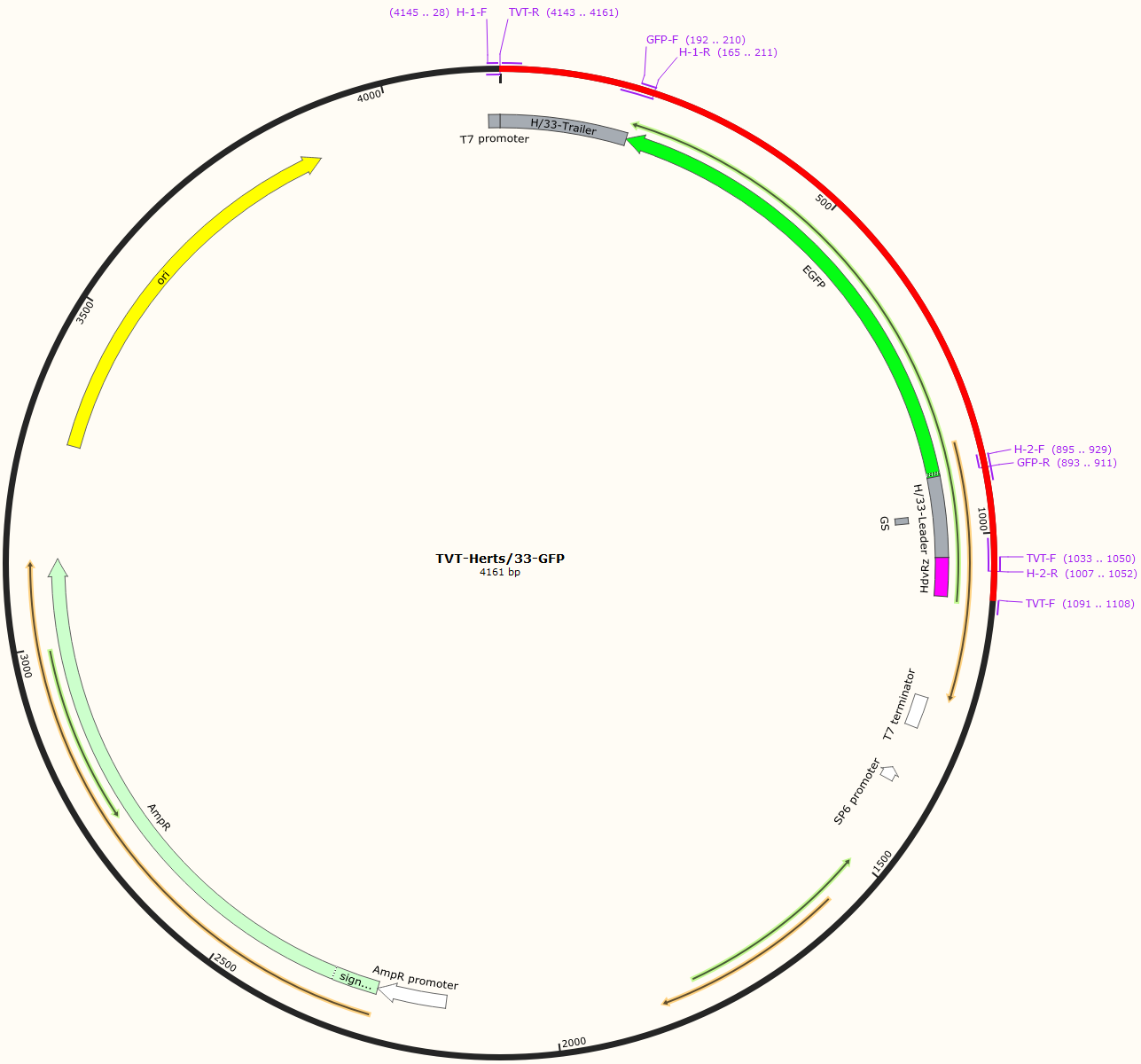

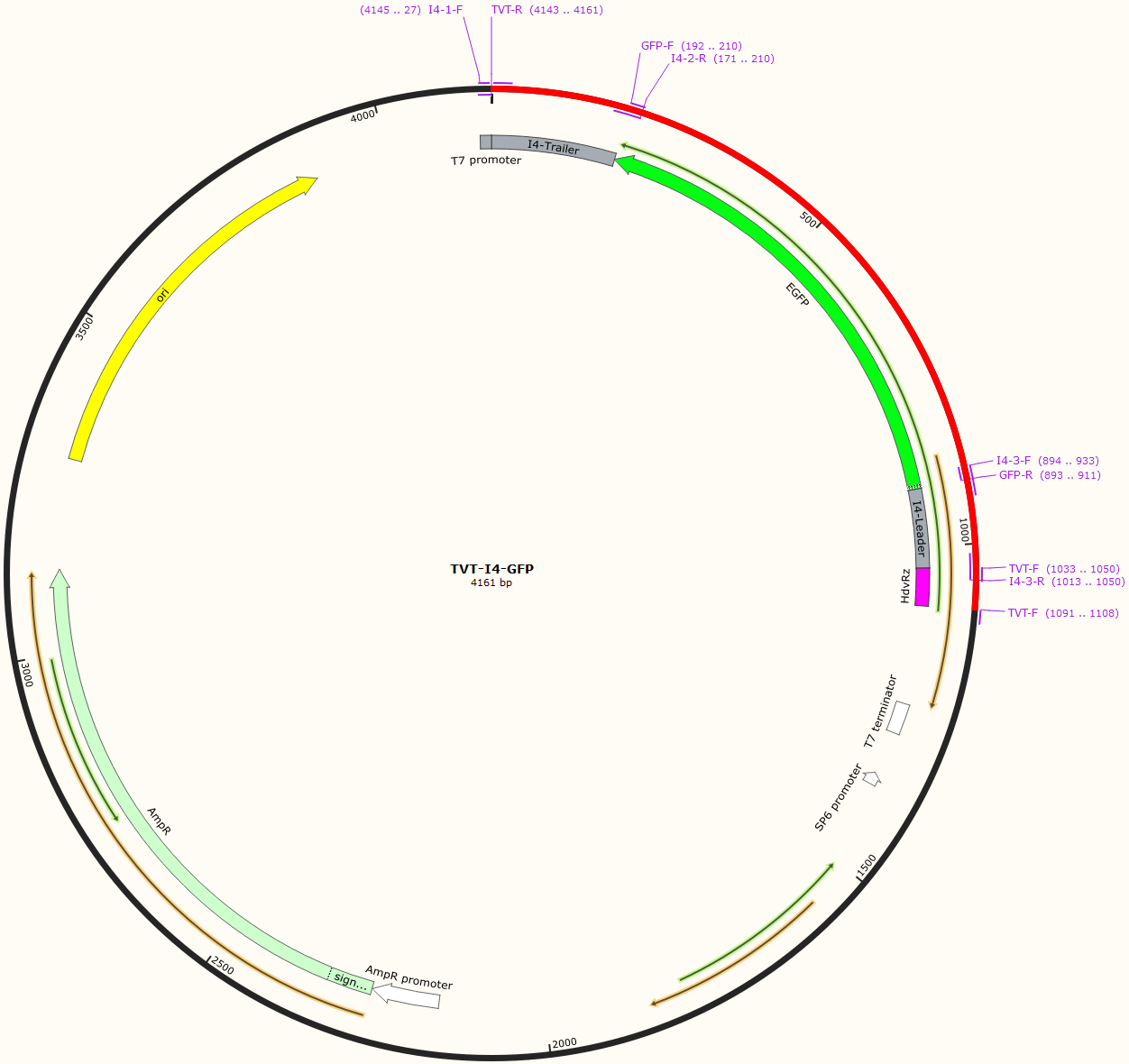

Supplement: S2 Table — (DOCX) [file ppat.1012027.s002.docx]
