# Supplementary material for: The NP protein of Newcastle disease virus dictates its oncolytic activity by regulating viral mRNA translation efficiency
Source: PLoS Pathog. 2024 Feb 20;20(2):e1012027. doi: 10.1371/journal.ppat.1012027 (PMC10906838; doi:10.1371/journal.ppat.1012027)
Supplement: S3 Table — (DOCX) [file ppat.1012027.s003.docx]

**S3 Table. Primer sequences for the plasmids using the pCAGGS vector**

| Application | Primer | Sequence (5’-3’) |
| --- | --- | --- |
| pCAGGS-H-NP-His | HNP- F | CATTTTGGCAAAGAATTCATGTCTTCCGTATTCGACGAATACGAG |
|  | HNP- R | CTAGCTCGAGCATGCCCGGGTCAATGGTGATGGTGATGATGATACCCCCAGTCGGTGT |
| pCAGGS-H-NP_1-245_-His | HNP- F | as mentioned above |
|  | HNP_1-245_-R | TCGAGCATGCCCGGGTCAATGGTGATGGTGATGATGGCTCCCACCTGCCGTG |
| pCAGGS-H-NP_245-366_-His | HNP_245-489_F | CTCATCATTTTGGCAAAGAATTCATGTCCACCTATTACAACTTGGTAGGG |
|  | HNP- R | as mentioned above |
| pCAGGS-H-NP_1-366_-His | HNP- F | as mentioned above |
|  | HNP_1-366_-R | CTCGAGCATGCCCGGGTCAATGGTGATGGTGATGATGAGCATACTCTACTCCAAGTC |
| pCAGGS-H-NP_366-489_-His | NP_367-489_-F | TCTCATCATTTTGGCAAAGAATTCATGCAGGCTCAGGGAAGTAGC |
|  | HNP- R | as mentioned above |
| pCAGGS-H-NP_122-366_-His | HNP-122-489-F | CATTTTGGCAAAGAATTCATGAGAGCACAGAGATTCTTGATGAT |
|  | HNP_1-366_-R | as mentioned above |
| pCAGGS-I-NP-His | Pcaggs-I4-NP-His-F | CATCATTTTGGCAAAGAATTCATGTCGTCTGTTTTCGACGAATACG |
|  | Pcaggs-I4-NP-His-R | CTAGCTCGAGCATGCCCGGGTCAATGGTGATGGTGATGATGGTACCCCCAGTCAGTGTCGT |
| pCAGGS-eIF4A1-HA | pCAGGS-eIF4A1-HA-F | TCATCATTTTGGCAAAGAATTCatgtctgcgagccaggattc |
|  | pCAGGS-eIF4A1-HA-R | atgttgctgacctcatcTACCCATACGATGTTCCAGATTACGCTtagGCTAGCAGATCTTTTT |

Note: The red sequence represents the His tag sequence. The green sequence represents the Ha tag sequence.
