# Supplementary material for: The NP protein of Newcastle disease virus dictates its oncolytic activity by regulating viral mRNA translation efficiency
Source: PLoS Pathog. 2024 Feb 20;20(2):e1012027. doi: 10.1371/journal.ppat.1012027 (PMC10906838; doi:10.1371/journal.ppat.1012027)
Supplement: S4 Table — (DOCX) [file ppat.1012027.s004.docx]

**S4 Table. Primers sequences used for the construction of plasmids in the BiFC experiment**

| Application | Primer | Sequence (5’-3’) |
| --- | --- | --- |
| VC155-EIF4A1 | VC155-EIF4A1-F | **TATGGCCATGGAGGCCCGAATT**atgtctgcgagccaggattc |
|  | VC155-EIF4A1-R | **GCACGCCGGACGGGTACCTCGAGG**gatgaggtcagcaacattga |
| VN173-HNP | VN173-HNP-F | **ACGACAAGCTTGCGGCCGCGAATTCA**ATGTCTTCCGTATTCGA |
|  | VN173-HNP-R | **ACCATGGTGGCGATGGATCTTCTAGA**ATACCCCCAGTCGGTGTCA |
| VN173-INP | VN173-INP-F | **CAAGCTTGCGGCCGCGAATTCA**ATGTCGTCTGTTTTCGACGA |
|  | VN173-INP-R | **CATGGTGGCGATGGATCTTCTAGA**GTACCCCCAGTCAGTGTC |

Note: The homologous arms are marked in bold.
