# Supplementary material for: The NP protein of Newcastle disease virus dictates its oncolytic activity by regulating viral mRNA translation efficiency
Source: PLoS Pathog. 2024 Feb 20;20(2):e1012027. doi: 10.1371/journal.ppat.1012027 (PMC10906838; doi:10.1371/journal.ppat.1012027)
Supplement: S5 Table — (DOCX) [file ppat.1012027.s005.docx]

**S5 Table. Primer and probe sequences used in the qRT-PCR experiments**

| Application | Primer/Probe | Sequence (5’-3’) |
| --- | --- | --- |
| NDV-NP | NP-qPCR-F | GACCAGATGAGCTTTGCACCA |
|  | NP- qPCR-R | AATGATGTGCTCATAAAGT |
|  | Probe | TAGAGTATGCTCAGGCTC |
| NDV-HN | HN-qPCR-F | AGTGATGTCACATCATT |
|  | HN- qPCR-R | GAAGCACACCAAGTGCTAAG |
|  | Probe | GGTATTCTTTTCTACTCTGC |
| GFP | F | CGACAAGCAGAAGAACGGCATCA |
|  | R | GGACTGGGTGCTCAGGTAGTGGTT |
|  | Probe | CGTTGTGGCTGTTGTAGT |
| IFN-α | F | cttgggatgaggacctcctagac |
|  | R | gcacaagggctgtatttcttctc |
|  | Probe | ttgtcagagcagaaatca |
| IFN-β | F | tgggaggcttgaatactgcctcaa |
|  | R | tcattccagccagtgctaga |
|  | Probe | gttgagaacctcctggctaat |
| Myc | F | agcagcagcagcagagcgag |
|  | R | gaagctcccgccaccgccgt |
|  | Probe | gagatggtgaccgagctgct |
| cyclin A2 | F | ccaacaaccgcggacccggg |
|  | R | ggtgaacgcaggctgtttac |
|  | Probe | aagaagctcagaagaagcc |
| cyclin B1 | F | aatatgtgaaagatatttat |
|  | R | tggagacagtcatgtacatg |
|  | Probe | atgcagaataattgtgtgcc |
| β-actin | F | gtttgatgatggggcatagg |
|  | R | ctccttgggagccacaact |
|  | Probe | ccaaggccaaccatgag |
