# Supplementary material for: The NP protein of Newcastle disease virus dictates its oncolytic activity by regulating viral mRNA translation efficiency
Source: PLoS Pathog. 2024 Feb 20;20(2):e1012027. doi: 10.1371/journal.ppat.1012027 (PMC10906838; doi:10.1371/journal.ppat.1012027)
Supplement: S6 Table — (DOCX) [file ppat.1012027.s006.docx]

**S6 Table. Primer sequences for the construction of NP protein prokaryotic expression plasmid**

| Application | Sequence (5’-3’) |
| --- | --- |
| pET-HNP-F | **TCCGCGTGGATCCAAGCTT**ATGTCTTCCGTATTCGA |
| pET-HNP-R | **TCTAGGCTAAAACCTCGAATTC**TCAATACCCCCAGTCGGTGT |
| pET-INP-F | **CTGGTTCCGCGTGGATCCAAGCTT**ATGTCGTCTGTTTTCG |
| pET-INP-R | **TCTAGGCTAAAACCTCGAATTC**TCAGTACCCCCAGTCAGTGT |

Note: The homologous arms are marked in bold.
