# Supplementary material for: The NP protein of Newcastle disease virus dictates its oncolytic activity by regulating viral mRNA translation efficiency
Source: PLoS Pathog. 2024 Feb 20;20(2):e1012027. doi: 10.1371/journal.ppat.1012027 (PMC10906838; doi:10.1371/journal.ppat.1012027)
Supplement: S7 Table — (DOCX) [file ppat.1012027.s007.docx]

**S7 Table. The sequence of siRNA used in this paper**

| Application | Sequence (5’-3’) | Sequence (5’-3’) |
| --- | --- | --- |
| RIG-I（homo） | AAUUCAUCAGAGAUAGUCATT | UGACUAUCUCUGAUGAAUUTT |
| PKR （homo） | GCGAGAAACUAGACAAAGUTT | ACUUUGUCUAGUUUCUCGCTT |
| eIF4A1  （homo） | CUGGCCGUGUGUUUGAUAUTT | auaucaaacacacggccagTT |
