# Supplementary material for: The NP protein of Newcastle disease virus dictates its oncolytic activity by regulating viral mRNA translation efficiency
Source: PLoS Pathog. 2024 Feb 20;20(2):e1012027. doi: 10.1371/journal.ppat.1012027 (PMC10906838; doi:10.1371/journal.ppat.1012027)
Supplement: S8 Table — (DOCX) [file ppat.1012027.s008.docx]

**S8 Table. Primers for generating HeLa cell lines with stable expression of the NP protein**

| Primer | Sequence (5’-3’) |
| --- | --- |
| pWPXL-HNP-F | AGGTTTAAACTACGGGATCCATGTCTTCCGTATTCGACGAATAC |
| pWPXL-HNP-R | ATATGACTAGTCCCGGGAATTCTCAATACCCCCAGTCGGTGTCA |
| pWPXL-INP-F | AGGTTTAAACTACGGGATCCATGTCGTCTGTTTTCGACGAATAC |
| pWPXL-INP-R | TATGACTAGTCCCGGGAATTCTCAGTACCCCCAGTCAGTGTCGTT |
