# Supplementary figures and images for: The NP protein of Newcastle disease virus dictates its oncolytic activity by regulating viral mRNA translation efficiency
Source: PLoS Pathog. 2024 Feb 20;20(2):e1012027. doi: 10.1371/journal.ppat.1012027 (PMC10906838; doi:10.1371/journal.ppat.1012027)

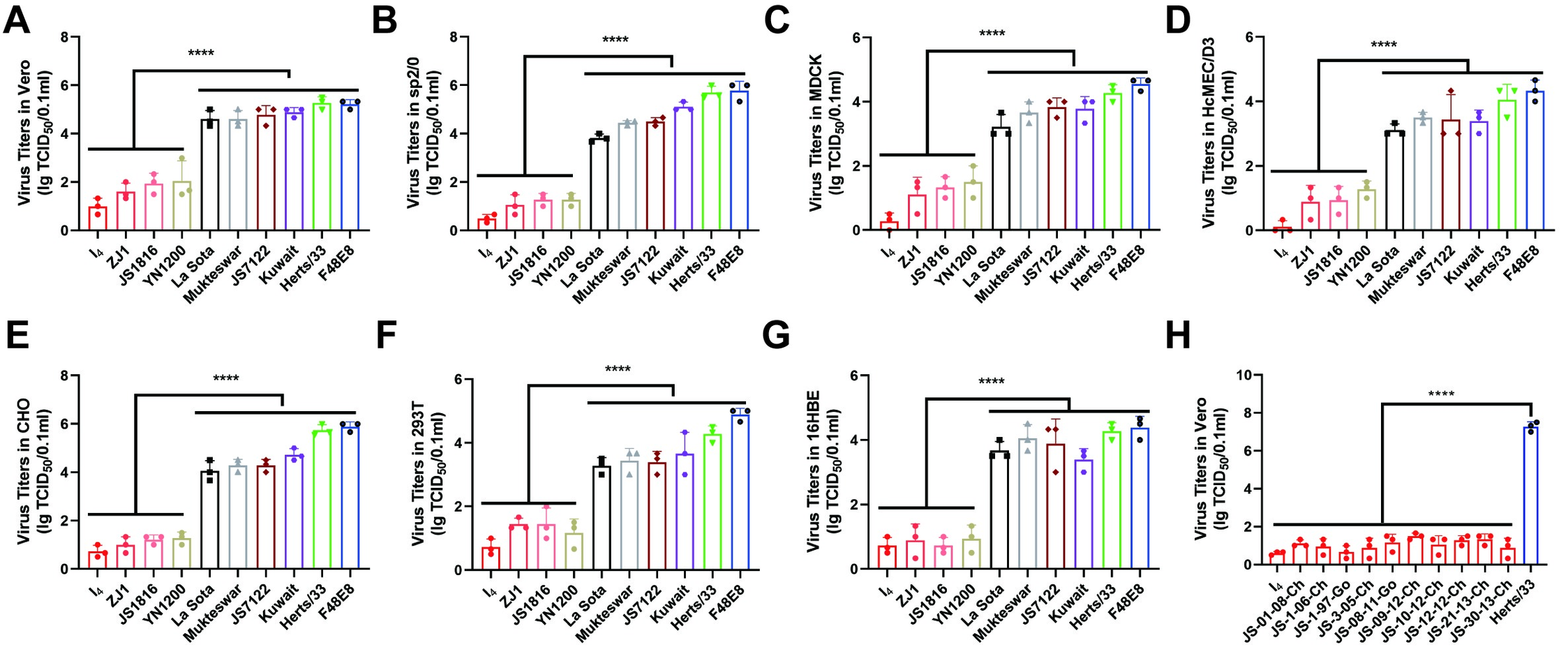

Supplement: S1 Fig — Viral titers at 72hpi in (A) Vero, (B) sp2/0, (C) MDCK, (D) HcMEC/D3, (E) CHO, (F) 293T, (G) 16HBE, (H) TCID50 value of ten additional genotype VII NDV strains in HeLa cells. Representative data, shown as the mean ± SD (n = 3), were analyzed with one-way ANOVA. ****, P<0.0001. (TIF) [file ppat.1012027.s009.tif]

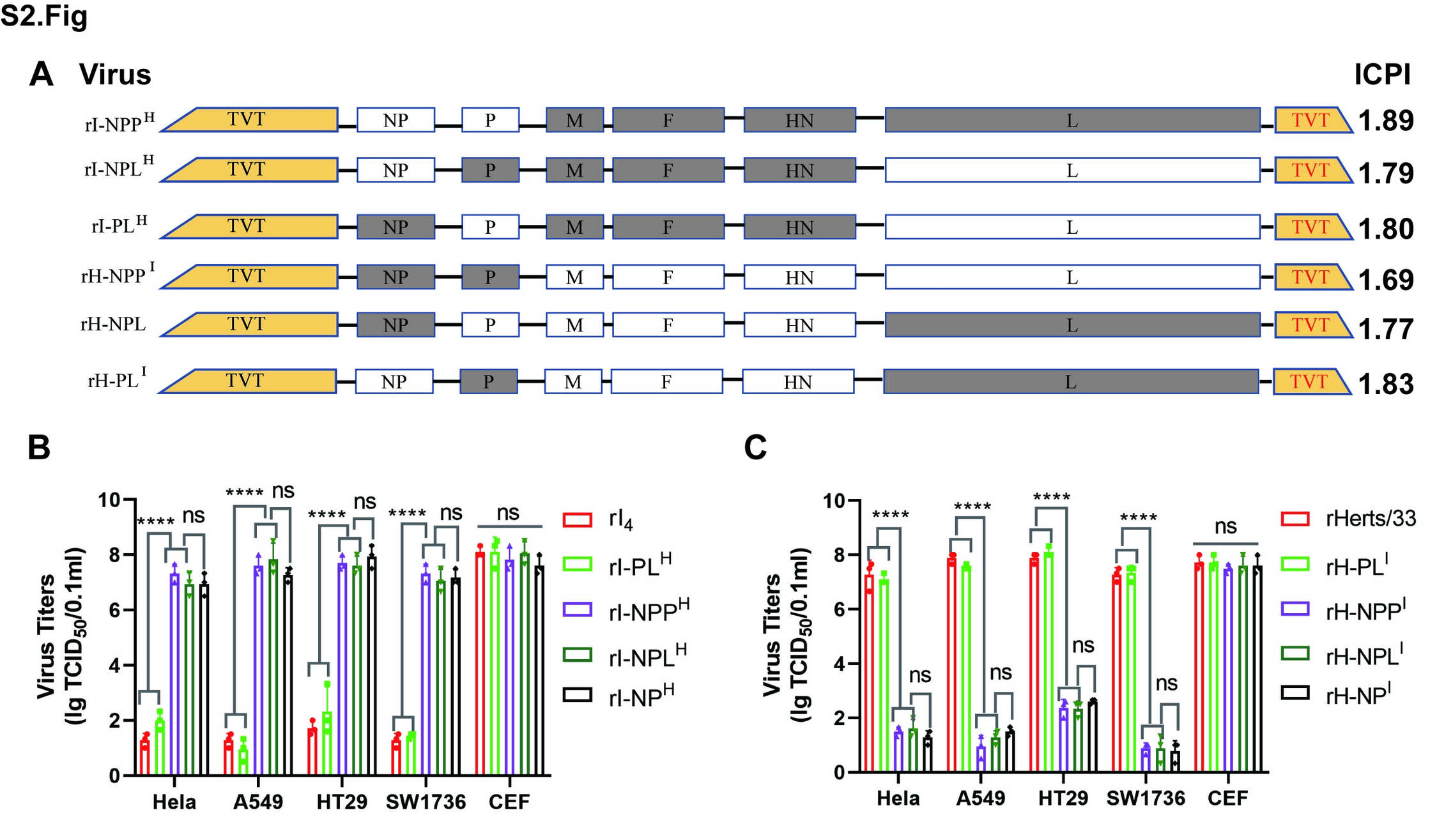

Supplement: S2 Fig — (A) The cloning strategy schematic for simultaneous replacement of NP and P, NP, and L, or P and L genes between rHerts/33 and rI4. The virulence of the different recombinant viruses was determined by measuring the Intracerebral Pathogenicity Index (ICPI) in 1-day-old chickens. (B and C) TCID50 value of the virulent strains after simultaneous replacement of NP and P, NP and L, or P and L genes. Representative data, shown as the means ± SDs (n = 3), were analyzed with two-way ANOVA. ****, P<0.0001. (TIF) [file ppat.1012027.s010.tif]

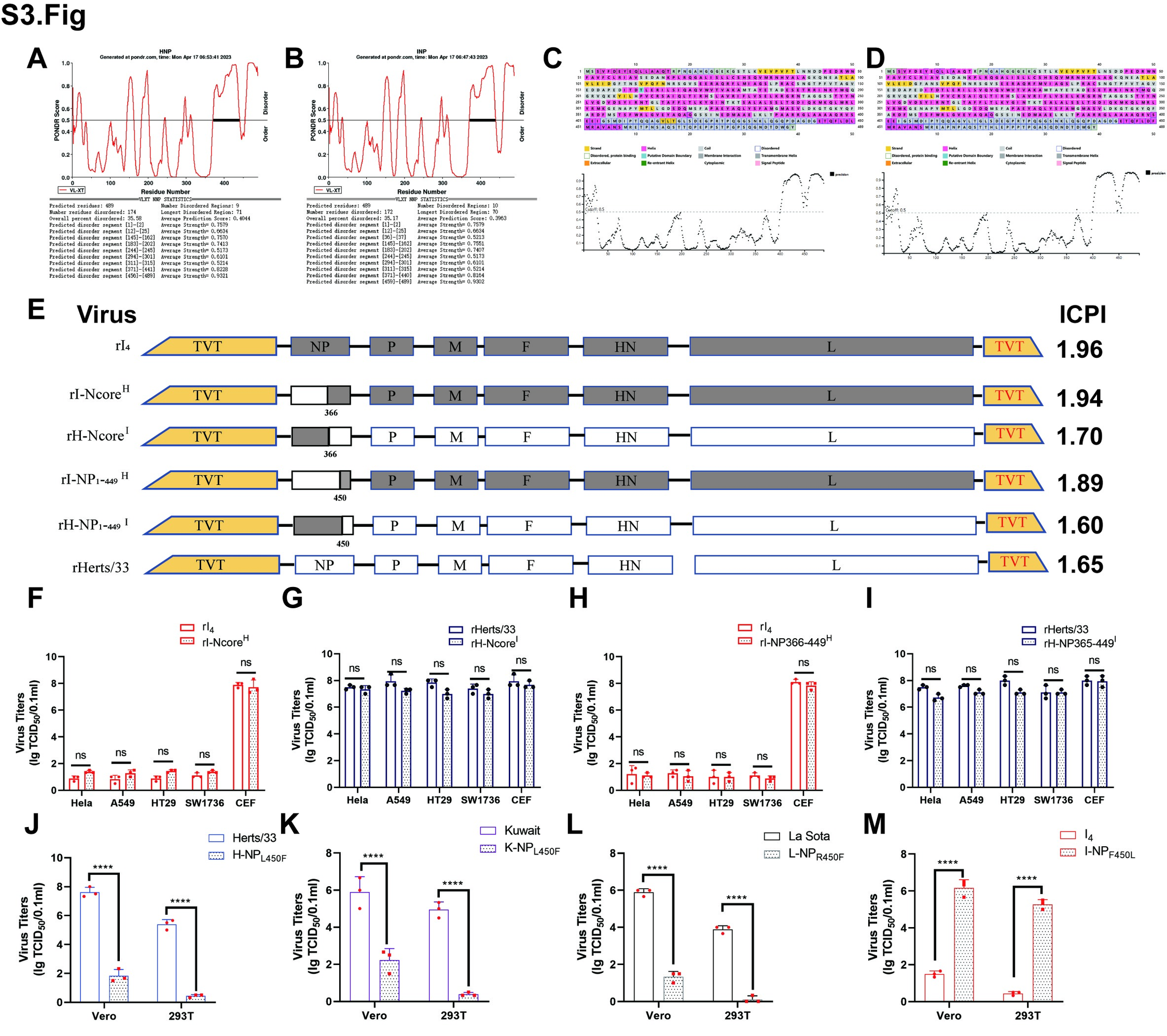

Supplement: S3 Fig — (A and B) PONDR was used to predict the IDR of HNP (A) and INP (B) (http://www.pondr.com/). VL3 Predictor (Developed by P. Radivojac and A.K. Dunker) was used. Regions with a score greater than 0.5 are considered disordered regions. (C and D) PSIPRED was used to predict the IDR of HNP (A) and INP (B) (http://bioinf.cs.ucl.ac.uk/psipred/?disopred=1). Regions with a score greater than 0.5 are considered disordered regions. (E) Schematic diagram of the cloning strategy for exchanging the whole N-core and the first IDR of the N-tail domain between rHerts/33 and rI4. The virulence of the different recombinant viruses was determined by measuring the ICPI in day-old chickens. (F, G, H, and I) TCID50 value of recombinant strains after replacement of the whole N-core and the first IDR of N-tail domain. (J, K, L, M) TCID50 values of mutant NDVs at 72hpi on several non-tumor cell lines. Representative data, shown as the means ± SDs (n = 3), were analyzed with two-way ANOVA. ****, P<0.0001. (TIF) [file ppat.1012027.s011.tif]

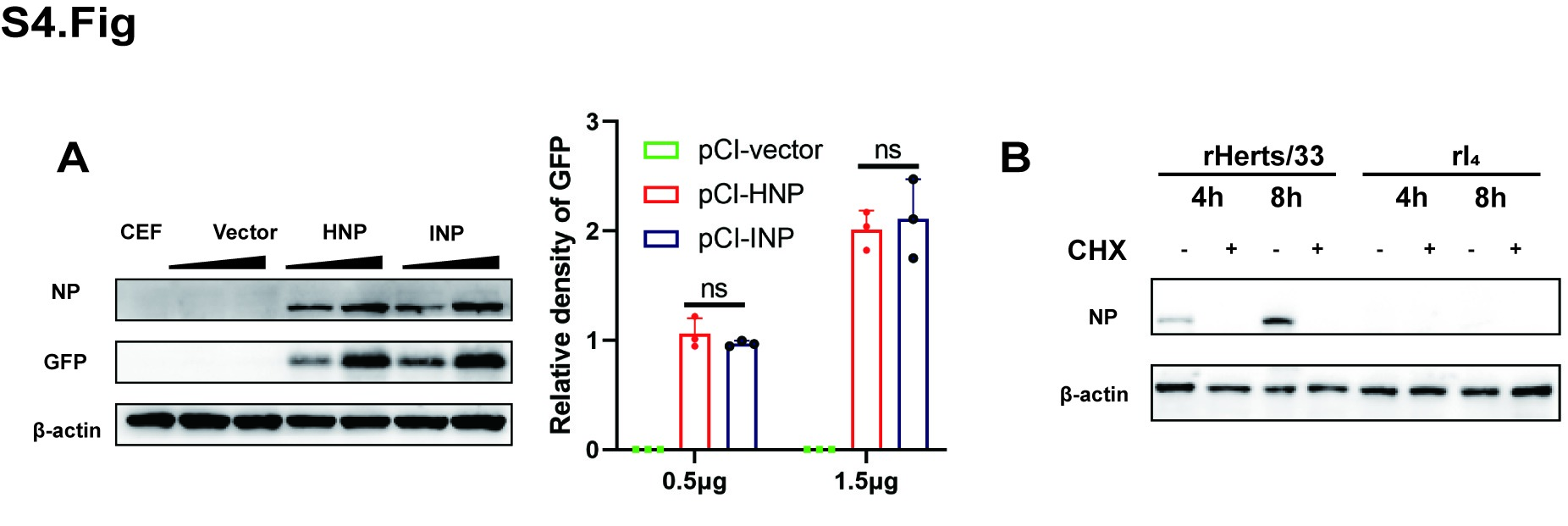

Supplement: S4 Fig — (A) Expression of GFP was detected at 24 h in CEF cells after transfecting 0.5 μg or 1.5 μg minigenome with anti-GFP, anti-NP, and anti-β-actin. (B) HeLa cells were treated with 100μg/ml CHX for 1h and then infected with NDV (10MOI) at 37°C for 0.5h. After that, cells were collected at 0h, 1h, 2h, 4h, and 8h. (TIF) [file ppat.1012027.s012.tif]

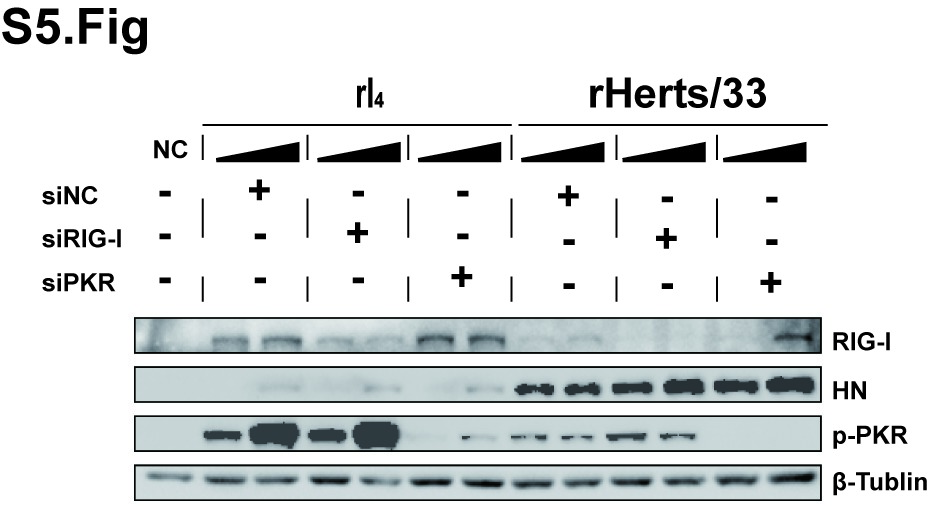

Supplement: S5 Fig — HeLa cells were transfected with control siRNA or specific siRNA targeting RIG-I and PKR. After 36 h, cells were infected with NDV at 1 MOI or 10 MOI and collected at 24 hpi for analysis by immunoblotting with anti-RIG-I, anti-HN, anti-p-PKR, anti-PKR, or anti-β-actin antibodies. (TIF) [file ppat.1012027.s013.tif]

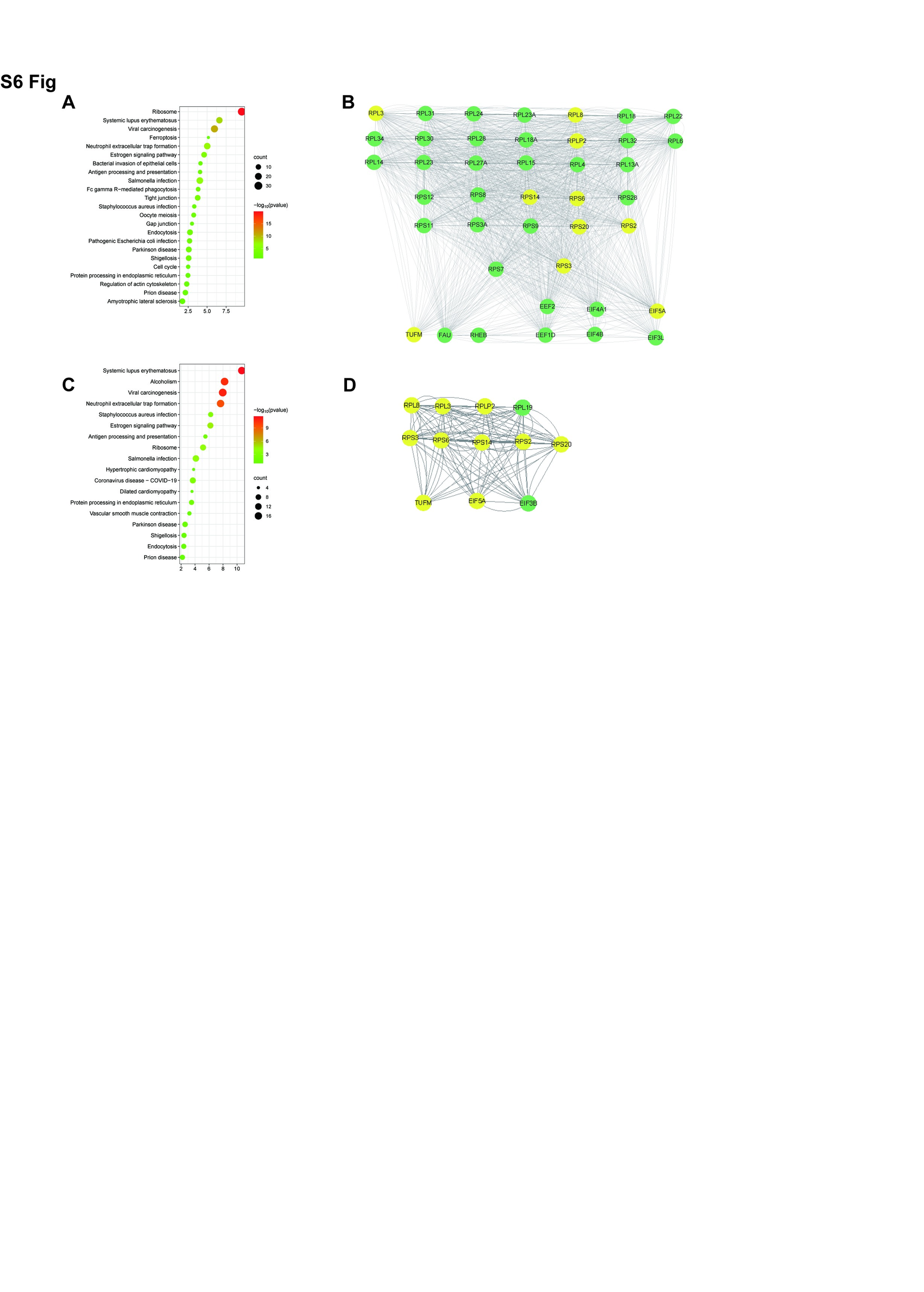

Supplement: S6 Fig — Pathway analysis of the cellular proteins interacting with HNP (A) and INP (C) based on KEGG were performed using KEGG analysis tool in the Database for Annotation Visualization and Integrated Discovery (DAVID) (version 6.7). The Protein-Protein interaction networks of the cellular proteins interacting with HNP (B) and INP (D) was constructed using the Cytoscape software. Yellow nodes represent proteins specific to each interaction partner, while green nodes represent proteins that interact with both HNP and INP. (TIF) [file ppat.1012027.s014.tif]

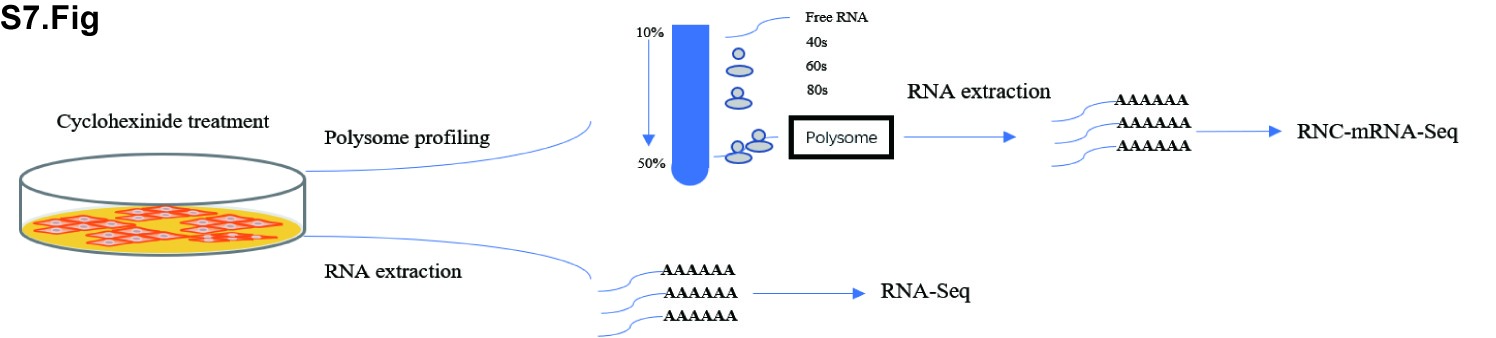

Supplement: S7 Fig — HeLa cells stably expressing different sources of NP and control HeLa cells were used to isolate ribosomes and enrich mRNA for multimeric fractions for RNC-Seq, and the other half were directly extracted for total RNA and enriched mRNA for RNA-Seq. (TIF) [file ppat.1012027.s015.tif]
