# Supplementary material for: The NP protein of Newcastle disease virus dictates its oncolytic activity by regulating viral mRNA translation efficiency
Source: PLoS Pathog. 2024 Feb 20;20(2):e1012027. doi: 10.1371/journal.ppat.1012027 (PMC10906838; doi:10.1371/journal.ppat.1012027)
Supplement: Attachment — Submitted filename: response to reviewers.docx [file ppat.1012027.s016.docx]

**Dear Editor and Reviewers,**

**We sincerely appreciate your patient guidance and valuable suggestions on our manuscript. These recommendations have been immensely helpful to us. We carefully reviewed the suggestions and have made revisions and additions to our manuscript accordingly. Supplementary experimental results have been included in Figures 8 and 9, primarily in response to the first reviewer's suggestions. Despite our best efforts to enhance our findings, we acknowledge that we have not fully addressed the first reviewer's request, which stated, "The authors should investigate the mechanism of the defectiveness of NPs with F450 in activating viral mRNA translation in mammalian cells. The previous work (PMID: 32603377) may provide some useful guidance." Indeed, previous work (PMID: 32603377) has provided us with valuable insights. We are diligently working in this direction, but it may still require one to two years to achieve significant progress. We are grateful for the various experimental method suggestions provided by the second reviewer. These were issues that we either overlooked or were unclear about. We have repeated all experiments related to these questions using the correct methodologies and updated the results.** **Due to numerous revisions, we sincerely appreciate the editors and reviewers for taking the time to review our revised manuscript. In this letter, we have highlighted all the experts' suggestions in yellow and provided responses in gray. For ease of reference, we have indicated the line numbers where modifications are made in the clean manuscript in our gray responses.**

**We highly appreciate the opportunity you have given us to submit the revised manuscript. Thank you very much for your guidance and valuable recommendations.**

**Sincerely,**

**Prof. Dr. Xiufan Liu**

**Yangzhou University, Yangzhou, China**

**E-mail:** [**xfliu@yzu.edu.cn**](mailto:xfliu@yzu.edu.cn)

**Part I - Summary**
Please use this section to discuss strengths/weaknesses of study, novelty/significance, general execution and scholarship.

Reviewer #1: Liao and colleagues investigated an unexpected observation that the replication of virulent NDV strains belonging to genotype VII is severely restricted in multiple human cancer cell lines. Productive NDV replication is highly specific to avian hosts, and it is generally believed that the major restriction factor in mammals is the inefficient inactivation of the interferon response by the viral protein V. This mediates the selective replication of NDVs in cancer cells with defects in anti-viral signaling making them promising oncolytic agents. In this case, however, the authors by making chimeric constructs between the NDV strain effectively replicating in cancer cell lines (Herts/33) and the representative of the genotype VII (I4) narrowed down the restriction determinant to amino-acid 450 in the nucleocapsid protein (NP), which is F in genotype VII (replication restricted) and L in other genotypes (replication-competent). Further analysis using polysome profiling and minigenome replication systems revealed that NP with L450 was associated with polysomes at a significantly higher level than F450, and accordingly, more GFP-coding mRNA generated by the minigenome system was found in the polysomal fraction with NP with L450. The authors also identified that amino-acids 122-366 of NPs contain the domain required for the association of NP with polysomal fraction. Altogether, the presented data show that NPs with L450 are competent in promoting translation of virus-generated mRNAs in mammalian cells, while those with F450 are not.
The observation of a specific NP-mediated restriction of genotype VII replication in mammalian cells is interesting, but the mechanistic insights are insufficient. The role of NDV NPs in promoting virus-specific mRNA translation has been reported before (PMID: 32603377) and was attributed to the interaction of NP with the translation initiation factor eIF4E. Also, the data on the presumable specific inhibition of translation of IFN-coding mRNAs by the NP with L450 (from replication-competent viruses) are overinterpreted:

Fig. 7. The results reflect the differences of the I4 and Hert 33 replication, leading to a stronger general suppression of host mRNA translation, not a preferential inhibition of IFN mRNA translation in the latter case
Response: Dear reviewer, thank you for your valuable suggestions. You are absolutely right, the rapid replication of Herts/33 leads to the overall inhibition of host mRNA translation. In response to your suggestions, we have supplemented the mRNA levels of β-actin detected in the ribosomal components. To better align with the central focus of our study, we have made revisions to the conclusion and the description in Figure 7. Specifically, we have rephrased it to indicate that the translation efficiency of viral mRNA in NDV with the 450th-L-NP phenotype is higher than that in NDV with the 450th-F-NP phenotype. However, it's important to note that the translation efficiency of cellular mRNAs, including Type I IFN and β-actin, in cells infected with the 450th-L-NP phenotype NDV is significantly lower compared to what we observe in the 450th-F-NP phenotype NDV. As a result, we have concluded that the 450th-L-NP promotes the loading of viral mRNA onto ribosomes during the infection process (Fig 7, line 691).

Fig. 7E qPCR data only show the amount of NP-coding mRNA, these data can not be extrapolated to all virus-generated mRNAs and should be described accordingly (line 600)

Response: Dear reviewer, thank you for your valuable suggestions. In this section, we have supplemented the mRNA detection results of the NDV HN gene. The relevant experiments have been repeated, and modifications have been made to the results. （Fig 7, line 690-691）

Reviewer #2: In this manuscript, the authors discovered that the 450th amino acid of the NP protein is critical for the oncolytic activity of NDV strains. Their subsequent experiments revealed that amino acid differences in the 450th position of NP affected its binding efficiency with mammalian polysomes. Their finding is highly suggestive, as it answers a long-standing question about why the oncolytic activity of NDV appears to be unrelated to its virulence. The results explain this phenomenon from the perspective of host selective translation and have great significance in guiding research on oncolytic viruses. There is a great deal of work here. The authors are certainly to be commended for their effort in telling a complete story. They present a fairly strong case for the conclusions listed above. However, there are a number of significant issues that should be addressed in a revised version, as well as numerous minor points.

**Part II – Major Issues: Key Experiments Required for Acceptance**
Please use this section to detail the key new experiments or modifications of existing experiments that should be absolutely required to validate study conclusions.

Generally, there should be no more than 3 such required experiments or major modifications for a "Major Revision" recommendation. If more than 3 experiments are necessary to validate the study conclusions, then you are encouraged to recommend "Reject".

Reviewer #1: The authors should investigate the mechanism of the defectiveness of NPs with F450 in activating viral mRNA translation in mammalian cells. The previous work (PMID: 32603377) may provide some useful guidence

Response: Dear reviewer, thank you for your patient guidance. Over the past two months, we have endeavored to provide a more comprehensive investigation into the mechanistic aspects from the perspective of differential interactome proteins. To this end, we employed mass spectrometry sequencing to identify differential interactome proteins of the NP protein from highly infectious Herts/33 (HNP) and the poorly infectious I_4_ strain (INP). We observed an interaction between eIF4A1 and HNP spanning amino acids 366-489, and it was found that mutations within the 450th amino acid position did not affect the interaction between HNP and eIF4A1. Notably, eIF4A1 did not interact with INP. Moreover, inhibiting the expression of eIF4A1 slightly enhanced NDV replication, suggesting a relatively simple 5'UTR region structure in NDV mRNA, and eIF4A1 independence in the translation of NDV mRNA. Moreover, HNP was discovered to suppress the translation of host mRNA that relies on eIF4A1, whereas INP lacks this capability, thus presenting an alternative mechanism by which HNP enhances viral replication. This also elucidates why the mutant strain with a leucine substitution at the 450th phenylalanine residue of the I_4_ strain could not fully match the replication capability on tumor cells as observed with Herts/33 （Fig 3I）. This is because HNP has other structural domains that play vital roles in viral replication. To summarize, our paper can be distilled into three key conclusions:

1. NP protein functions as a differentiating factor in the distinct replication abilities of highly infectious Herts/33 and poorly infectious I_4_ on tumor cells.

2. The 450th amino acid position of HNP has a substantial effect on the loading of viral mRNA onto ribosomes. (However, we apologize for not currently providing a molecular explanation for this conclusion's core mechanism from a cell biology perspective. The 450th amino acid of HNP may play a crucial role in the recognition and translation initiation of viral mRNA in host cells, given that NDV mRNA lacks 2'-O-methylation compared to eukaryotic mRNA. Our laboratory continues to make relevant efforts, but this may take several years of research.)

3. HNP interacts with cellular eIF4A1 from amino acids 366 to 489, inhibiting the host cell's eIF4A1-dependent translation activity, thereby facilitating the virus's increased utilization of cellular resources for translating viral mRNA. (Inhibition of eIF4A1-dependent mRNA translation by HNP may serve as a secondary inducer for the preferential translation of viral mRNA by HNP. We have also discovered that HNP can interact with various helicases, including DDX3, DDX19A, and DHX9, and related work is still in progress, so these data have not been presented or discussed in the article. We hypothesize that HNP has many unexplored roles in regulating host cell mRNA translation. The biological phenomenon we initially observed is, in fact, the extremely poor replication capability of multiple genotype VII NDV strains in various mammalian cells, including tumor cells. Therefore, the inability of the NP protein of genotype VII I_4_ to mediate efficient translation of viral mRNA in mammalian cells may be attributed to a significant divergence between translation-related proteins, including eIF4A1, in mammals compared to birds. This not only implies that most genotype VII NDVs, responsible for the fourth pandemic, is unsuitable as an oncolytic strain but also raises questions about its suitability as a vector vaccine for mammals, prompting considerations about the evolutionary direction of NDV. The related work is currently in progress. Below, we present the TCID_50_ results of different genotypic NDVs in seven non-tumor normal mammalian cells. The genotype VII strains are enclosed in red boxes.)

Reviewer #2: Line148-152: The cell infection of lentogenic NDV strains, like LaSota, requires extra addition of trypsin. It was not mentioned in the Methods. The concentration of trypsin in the medium is also a major factor affecting the infection efficiency of NDV strains in cells.
Response: Dear reviewer, we sincerely apologize for the elementary error made in the original text. In the subsequent description of the rescue methods for La Sota and its mutant strains, we provided an explanation regarding the concentration of TPCK trypsin (line 239). However, we inadvertently omitted this detail in the method description at line 171. For all infectivity experiments involving La Sota and its mutant strains in this study, we did indeed include TPCK trypsin to assist virus cell entry. Thank you for bringing this to our attention, and we have made the necessary correction. The relevant information is now located in line 171.

Line 236: there are some problems in the methods of virus binding assay. It is recommended that cells were incubated with NDV at 4℃ to prevent virus entry in the process of absorption.

Response:Dear reviewer, I appreciate your suggestions. We have repeated the virus binding experiment and conducted statistical analysis on the results. We have made revisions to the relevant methods section (line 265) and the results section (Fig 5， line 601).

Some of the author’s conclusions appear to lack rigor. For instance, the study only examined a limited number of genotype VII strains, yet the conclusion drawn was that genotype VII NDVs are not oncolytic. It is unclear whether this trait is specific to a group of strains or several genotypes. Furthermore, there is a possibility of genotype VII strains containing the F450L substitution in NP, which could provide oncolytic activity to these strains. The authors should carefully analyze the outcomes of their experiments and rigorously draw conclusions.
Response: Dear reviewer, I appreciate your guidance and valuable suggestions. Indeed, this section's conclusions lacked rigor. We have made revisions to the corresponding results and conclusions, such as in lines 22-23 of the abstract. We have changed the vague term "generic gene VII-type NDV" to " NDV strains with phenylalanine (F) at the 450th amino acid position of the NP protein (450th-F-NP)". Simultaneously, modifications have been made to the respective statements in lines 450 and 484.

The authors constructed a great number of recombinant viruses and plasmids, and their labelling lacks clarity, resulting the difficulty of understanding. Therefore, a more unambiguous and concise naming system is suggested for improved comprehension. Additionally, some strains have associated schematic diagrams, others do not. For instance, the rl-NPPLH and rH-NPPLL is the main research object in line 384-392, but their schematic diagrams were not displayed in Figure 1A and the background was not clear.
Response: Dear reviewer, I deeply appreciate your guidance and valuable suggestions. The scope of work presented in this manuscript is substantial. Commencing from the second conclusion, all parental strains or recombinant viruses were rescued using infectious full-length clone plasmids. Consequently, in all subsequent experiments, we have differentiated these strains by introducing an "r" prefix, such as rI_4_, rHerts/33, rI-NPPL^H^, and rH-NPPL^I^. In the context of the minigenome system, we adhered to prior research practices and denoted the transcription vectors containing the reverse genetic sequence for GFP as TVT-HGFP or TVT-IGFP. The gene sequences related to the polymerase, NP, P, and L, were cloned into the pCI-neo vector. Therefore, within the pertinent sections of Figures 5 and 6, we have substituted the previous simplified notation for HNP and INP-dominated minigenomes with the format pCI-HNP and pCI-INP. Additionally, sequences carrying HNP and its segments were cloned into the pCAGGS vector due to its superior expression efficiency compared to pCI-neo. Thus, in the corresponding sections of Figures 6, 8, 9, and 10, we have included the pCAGGS label. I extend my heartfelt apologies for any inconvenience this may have caused you. Furthermore, we have made revisions to the diagrams for rI-NPPL^H^ and rH-NPPL^H^ (line 488). This strain was constructed by other members of our laboratory and kindly provided to us (1)(line 488).

Figure 3J-M, it is recommended to detect and compare TCID50 titers of these strains on CEF.
Response: Dear reviewer, I appreciate your guidance and suggestions. We have incorporated a comparison of TCID_50_ values for the mutant strains on CEF in Figure 3J-M (line 541).

Line 472-487: it is suggested that the amino acid sequences of NP gene from classical oncolytic strains should be specially taken for alignment.
Response: Dear reviewer, thank you for your valuable suggestions. We have made an effort to include several NDV strains that have already entered clinical trials, such as F3aa, Hitchner B1, AMHA1, R2B, Ulster, Italien, AF2240, and Anhinga(2). The commonly used La Sota strain for clinical research was already present in our original figures and tables.（line 540, Fig 3H）

Line 499-500: According to the flow cytometry results in Figure 5A, the adsorption rate of rl4 is higher than that of rHerts/33. The flow cytometry assay should be repeated for no less than three times and the data of virus adsorption should be compared statistically.
Response: Dear reviewer, thank you for your valuable suggestions. Following your advice, we have made modifications to the experimental methods in the virus binding assay. Additionally, we have conducted statistical analysis on the experimental results. Corresponding revisions have been made to Figure 5A (line 600).

Line 505-517: Based on the WB assay shown in Figure 5H, it appears that the HNP protein decreases due to CHX treatment. The author should repeat this experiment no less than three times, scan the gray scale, and perform statistical analysis. In my opinion, the results here suggest that the functional difference between HNP and INP may lie in translation, since CHX is an inhibitor of translation elongation.
Response: Dear reviewer, thank you for your valuable suggestions. We have repeated this set of experiments multiple times and conducted statistical analysis. Corresponding revisions have been made to Figure 5H (line 600).

Figure 6A: RNase digestion is recommended here to determine whether NP or extra RNA is responsible for the difference in polysome amounts.
Response: Dear reviewer, thank you for your valuable suggestions. Given that in Figure 6A, our primary objective was to demonstrate higher detection of 450th-L-NP-dominated GFP mRNA in ribosome components, the addition of RNA enzymes could potentially affect our subsequent detection of GFP mRNA. Therefore, based on your suggestion, we have made adjustments to the experiments in Figure 6E, where we explore the domains of HNP protein binding to ribosomes. After cell lysis with a polyribosome lysis buffer, we introduced RNA enzymes followed by polyribosome separation.(Fig 6E, line 646）

In the RNC-seq assay, why did the authors use HNP and INP rather than 450aa mutants?
Response: Dear reviewer, replacing the entire HNP with I_4_ as the scaffold to generate recombinant viruses (rI-NP^H^) results in an infection level in mammalian cells comparable to that of rHerts/33. However, when the phenylalanine at position 450 of I_4_ is mutated to leucine, the mutant strain rI-NP_F450L_ exhibits lower infectivity in mammalian cells compared to rHerts/33. Your suggestion is absolutely on point. To provide a mechanistic explanation regarding the 450th amino acid, we should indeed utilize a mutant strain with the 450th amino acid alteration. Considering the substantial time required for conducting and supplementing the relevant experiments, we have opted for a modification in the overall manuscript direction. We now approach the entire text from two angles: first, we elucidate HNP's 450th amino acid promotes the loading of viral mRNA onto ribosomes, and second, we highlight HNP's interaction with eIF4A1 to inhibit the translation of host mRNA dependent on eIF4A1.

In all of the figures' bar graphs, the distinctions between the colors representing various samples are not clear. Thus, it is recommended to use colors that have more noticeable differences to present the data.
Response: Dear reviewer, thank you for your recommendations. We have made corresponding revisions to all figures throughout the manuscript. In doing so, we have employed colors that we believe offer clearer differentiation between various experimental groups.

Finally, the discussion is quite lengthy and aimless. It is advisable to restructure the language by clarifying the topic and removing any redundant information.

Response: Dear reviewer, thank you for your valuable suggestions. In the revised manuscript, we have streamlined and focused the discussion section for clarity.

**Part III – Minor Issues: Editorial and Data Presentation Modifications**
Please use this section for editorial suggestions as well as relatively minor modifications of existing data that would enhance clarity.

Reviewer #1: Lines 50-52. P and L are not structural proteins

Response: Dear reviewer, thank you for your valuable suggestion. The relevant portions of the manuscript have been amended to use the term "viral protein." This change has been made in accordance with your suggestion and is located in the revised manuscript at line 52-53.

Line 58. Replace “cellular activity” with cellular viability

Response: Dear reviewer, thank you for pointing out the issue. All of the “celluar activity” has been replaced by “celluar viability”. This modification has been made in the revised version at line. (line 442 and 444)

Line 74. Spell out MV (measles virus)?

Response: Dear reviewer, it is "Measles virus," abbreviated as "MeV." The corresponding section in the manuscript, located at line 76, has been appropriately revised. Thank you for pointing out the issue.

Line 86. Translation of viral mRNAs, not proteins. Information in RNA is being translated into protein.

Response: Dear reviewer, Thank you for pointing out our mistake. The corresponding section in the manuscript has been revised. This modification has been made at line 107 of the revised manuscript.

Line 89. Spell MeV (measles virus)?

Response: Dear reviewer, in line 94, what we intended to express is "measles virus (MeV)."

Line 351. Fix genotype number

Response: Dear reviewer, thank you for your valuable suggestion. There was an issue during the conversion to PDF. The corresponding section has now been fixed (line 433).

Line 362. Replace “cellular activity” with cellular viability

Response: Dear reviewer, thank you for your valuable suggestion. All of the “celluar activity” has been replaced by “celluar viability”. This modification has been made in the revised version at line. (line 442 and 444)

Line 420. Remove “typically”

Response: Dear reviewer, thank you for your valuable suggestion. The word "typically" has been removed from the manuscript. This change has been implemented at line 502 of the revised manuscript.

Lines 444-445. These are not mutations, these are differences in amino-acid composition of the NP proteins of the two strains

Response: Dear reviewer, thank you for your valuable suggestion. You are absolutely correct. We had a description error. The corresponding section in the manuscript has been modified to state, "conserved differential sites" This change has been made at line 526 of the manuscript.

Line 625. Change “tir” to tyr (tyrosine)

Response: Dear reviewer, thank you for your valuable suggestion. The description in question has been modified and relocated to the figure caption section (Fig 10), at line 824 of the manuscript.

Lines 647-648. It is not clear what did the authors mean by “both HNP... promote translational activity by targeting translationally active regions, but HNP can selectively regulate cellular mRNA translation”

Response: Dear reviewer, thank you for your valuable suggestion. The conclusion in the original manuscript may not have been expressed clearly. In the revised version, we primarily aim to substantiate, through RNA-Seq results, that HNP can inhibit eIF4A1-dependent translation. (line 809-811)

Reviewer #2: L23: “the remaining genotypes” is supposed to be “the other genotype” or “the genotypes of rest”
Response: Dear reviewer, thank you for your valuable suggestion. We have revised the description accordingly in lines 24-6 of the revised manuscript.

L56-57: Does the sentence mean “there is no correlation between the oncolytic properties of NDV and its virulence”?
Response: Dear reviewer, thank you for your valuable suggestion. We have reviewed the relevant research, and a more suitable way to describe this would be: "NDV strains that exhibit lower pathogenicity in avian species can also demonstrate potent oncolytic capabilities, facilitated by the presence of TPCK protease."

Line320: define “RNC-Seq” when the abbreviation was first used.
Response: Dear reviewer, thank you for your valuable suggestion. In the revised manuscript, the relevant section now includes the complete term "Ribosome Nascent-chain Complex sequencing" at line 420.

Line351: genotype what?
Response: Dear reviewer, thank you for your valuable suggestion. There was an issue during the conversion to PDF. The corresponding section has now been fixed (line 433).

Figure1A-F: lack detection time point.
Response: Dear reviewer, thank you for your valuable suggestion. In this study, the determination of TCID_50_ values was performed at 72 hours post-virus infection. The manuscript has been revised accordingly.(Fig 1, 2 and 3)

Line387: the word “compatible” is not correct here.
Response: Dear reviewer, thank you for your valuable suggestion. The same description has also been found in other studies(3). We are uncertain about the most suitable replacement for that word and would appreciate guidance from our reviewers.(The Viral Replication Complex Is Associated with the Virulence of Newcastle Disease Virus)

Line456: The meaning of this sentence is not clear.
Response: Dear reviewer, thank you for your valuable suggestion. The relevant section in the original manuscript has been revised. We aim to convey, "In conclusion, our findings suggest that the 450th amino acid residue of NP plays a crucial role in NDV's infection on tumor cell lines."(line 538-539)

Figure 6E: “Ploysomes” should be “Polysomes”.
Response: Dear reviewer, thank you for your valuable suggestion. The spelling error in Figure 6E has been corrected.(line 646)

In the whole manuscript, all the log10 should be written as lg.
Response: Dear reviewer, thank you for your valuable suggestion. All instances of "log10" in the revised manuscript have been changed to "lg."

The author used the words minigenome and microgenome interchangeably. Please use the same name.

Response: Dear reviewer, thank you for your valuable suggestion. We have corrected all instances of "microgenome" to "minigenome" in the revised manuscript. These changes have been made in lines 587, 589, 607, and 609.

REFERENCES

1.	Kai Y, Hu Z, Xu H, Hu S, Zhu J, Hu J, et al. The M, F and HN genes of genotype VIId Newcastle disease virus are associated with the severe pathological changes in the spleen of chickens. Virol J. 2015;12:133.

2.	Huang F, Dai CJ, Zhang YN, Zhao YQ, Wang YG, Ru GQ. Development of Molecular Mechanisms and Their Application on Oncolytic Newcastle Disease Virus in Cancer Therapy. Frontiers in Molecular Biosciences. 2022;9.

3.	Dortmans JC, Rottier PJ, Koch G, Peeters BP. The viral replication complex is associated with the virulence of Newcastle disease virus. J Virol. 2010;84(19):10113-20.
