# Supplementary material for: The NP protein of Newcastle disease virus dictates its oncolytic activity by regulating viral mRNA translation efficiency
Source: PLoS Pathog. 2024 Feb 20;20(2):e1012027. doi: 10.1371/journal.ppat.1012027 (PMC10906838; doi:10.1371/journal.ppat.1012027)
Supplement: Attachment — Submitted filename: response to reviewers.docx [file ppat.1012027.s017.docx]

**Dear Editor and Reviewers,**

**We sincerely appreciate your patient guidance and valuable suggestions on our manuscript. In response to the first reviewer's advice, we have incorporated additional results and figures relevant to this study, as discussed in our previous correspondence with the reviewer. Following the second reviewer's recommendations, we have rectified inappropriate language, particularly in the abstract, author summary, and discussion sections. Additionally, we have included a model illustrating the impact of the NDV NP protein on its oncolytic ability (Fig 11). We are grateful for all the experts' recommendations, which have proven beneficial for our subsequent writing and submission. In this letter, we have highlighted all the experts' suggestions in yellow and provided responses in gray. For ease of reference, we have indicated the line numbers where modifications are made in the clean manuscript in our gray responses.**

**We highly appreciate the opportunity you have given us to submit the revised manuscript. Thank you very much for your guidance and valuable recommendations.**

**Sincerely,**

**Prof. Dr. Xiufan Liu**

**Yangzhou University, Yangzhou, China**

**E-mail:** [**xfliu@yzu.edu.cn**](mailto:xfliu@yzu.edu.cn)

Part I - Summary

Please use this section to discuss strengths/weaknesses of study, novelty/significance, general execution and scholarship.

Reviewer #1: The authors did a great job improving the manuscript and I am willing to support its publication provided they will incorporate the figure they placed in the reponse to reviewers letter in the paper. It is not a good practice to provide data for reviewers only, if the authors think they are relevant to the work, they should be available to all readers of the paper.

Response: Dear reviewer, thank you for your valuable suggestions. Your guidance would contribute to enhancing our subsequent writing and submission. We've addressed the points discussed in our previous communication. In S1 Fig, we've incorporated a chart comparing the infectivity of various genotypic NDVs in non-tumor mammalian cells. In lines 435-437, we state our observation of replication impediments in multiple genotype VII NDV strains in non-tumor mammalian cells and the more pronounced variability in infectivity among different NDV strains in tumor cells. In S3 Fig, we depict the infection outcomes of NDV strains with a mutation at the 450th amino acid of the NP protein in non-tumor mammalian cells. Emphasized in lines 537-538 is that the 450th amino acid not only influences NDV infectivity in tumor cells but also affects infectivity in non-tumor mammalian cells. Discussed in lines 864-868 is the speculation that NDV strains with a phenylalanine at the 450th amino acid of the NP protein are unsuitable for clinical tumor treatment and may not serve as vector vaccines for mammals. Lastly, in lines 927-930, we propose that the inability of the NP protein of genotype VII I_4_ to mediate efficient translation of viral mRNA in mammalian cells may be attributed to a significant divergence between translation-related proteins, including eIF4A1, in mammals compared to birds..

Reviewer #2: The authors discovered that the 450th amino acid of the NP protein is crucial for the oncolytic activity of NDV strains in this manuscript. Following many strong tests, they revealed that amino acid variations in the 450th position of NP influenced its binding efficiency with mammalian polysomes. There is a lot of work to be done here,and their discovery perfect answer that why NDV's oncolytic function appears to be unrelated to its pathogenicity.

Following the initial round of revision,the corresponding authors fully responded to the two reviewers' comments and rectified the faults in the manuscript to make it easier to understanding.

Here i have two suggestions

1.Please recheck the manuscript again to avoid the language mistake.For example line 26 “450th-F-NP exhibits weaker functionality in this process.”may not suitable to be a complete sentence in the abstract.

Response: Dear reviewer, thank you for your valuable suggestions. We have made modifications to the relevant section in line 26 and carefully reviewed for similar errors in the subsequent text. We have rectified inappropriate descriptions and corrected grammatical errors without altering the meaning of the sentences, primarily focusing on the title, abstract, and author summary. Thank you for bringing this to our attention.

2. The conclusion can be substantially supported by the experimental findings, and the viewpoint is also highly original. However, there is lack of an easy-to-understand pattern diagram to help readers rapidly understand the article's main themes. Please make the manuscript more legible by add a schematic plot, if possible.

Response: Dear reviewer, thank you for your valuable suggestions. We have supplemented the model diagram illustrating the differential oncolytic capabilities of NDV mediated by the NP protein (Fig 11).

Fig 11. Proposed model of the differential oncolytic capability mediated by NP Protein in NDV strains. (A) Translation of oncolytic NDV (Herts/33) mRNA: i HNP(450th-L-NP) efficiently recruits ribosomes and interacts with eIF4A1 through its 366-489aa, ii The efficiency of viral mRNA lacking 2′-O-methylation modification loading onto ribosomes is higher. The translation of cellular mRNA with complex structures in the 5’UTR, dependent on eIF4A1, is inhibited. These two processes together resulting in low efficiency of cellular mRNA loading onto ribosomes and distribution in areas unrelated to ribosomes, iii Efficient translation of viral mRNA, iv Production of a large number of viral particles. (B) Translation of non-oncolytic NDV (I_4_) mRNA: i INP(450th-F-NP) has weak binding ability with ribosomes and cannot interact with eIF4A1, ii Cellular mRNA containing 2’-O-methylation modification is efficiently loaded onto ribosomes. The translation of cellular mRNA with a complex structure in the 5’UTR, dependent on eIF4A1, is unaffected. Few viral mRNA is loaded onto ribosomes and more viral mRNA is distributed in regions unrelated to ribosomes, iii Extremely low efficiency of viral mRNA translation, iv Production of a small number of viral particles.

Part II – Major Issues: Key Experiments Required for Acceptance

Please use this section to detail the key new experiments or modifications of existing experiments that should be absolutely required to validate study conclusions.

Generally, there should be no more than 3 such required experiments or major modifications for a "Major Revision" recommendation. If more than 3 experiments are necessary to validate the study conclusions, then you are encouraged to recommend "Reject".

Reviewer #1: (No Response)

Reviewer #2: (No Response)

Part III – Minor Issues: Editorial and Data Presentation Modifications

Please use this section for editorial suggestions as well as relatively minor modifications of existing data that would enhance clarity.

Reviewer #1: (No Response)

Reviewer #2: (No Response)

PLOS authors have the option to publish the peer review history of their article (what does this mean?). If published, this will include your full peer review and any attached files.

If you choose “no”, your identity will remain anonymous but your review may still be made public.

Do you want your identity to be public for this peer review? For information about this choice, including consent withdrawal, please see our Privacy Policy.

Reviewer #1: No

Reviewer #2: No
