# Supplementary material for: The NP protein of Newcastle disease virus dictates its oncolytic activity by regulating viral mRNA translation efficiency
Source: PLoS Pathog. 2024 Feb 20;20(2):e1012027. doi: 10.1371/journal.ppat.1012027 (PMC10906838; doi:10.1371/journal.ppat.1012027)
Supplement: Attachment — Submitted filename: Response to editors and reviewers.docx [file ppat.1012027.s018.docx]

Your suggestions：

NDV is a US Federal Select Agent, and so your excellent work is considered potential DURC. The committee notes that the paper includes NDV variants that are (as you show) virulent, but also notes that the experiments are focused on tumor killing and not on infectivity or virulence of the NDV variants. The main concern is that the paper currently lacks any information about biosafety and does not describe institutional oversight and approval of the research. Therefore, please add a section to Materials and Methods that describes procedures and facilities for biosafety, in detail. In addition, please name the institutional committee(s) responsible for approval and oversight of the research.

Our response：

**Dear Editors and Reviewers,**

**We genuinely appreciate your patient guidance and valuable suggestions on our manuscript. All strains mentioned in the article were exclusively employed for studying NDV infectivity in tumor cells. Our findings highlight the crucial role of the 450th amino acid in NDV's NP protein during infection of tumor cells. Notably, NDV strains with leucine at the 450th amino acid exhibit oncolytic capabilities, whereas those with phenylalanine at this position show diminished oncolytic potential, primarily observed in genotype VII NDV. However, even when the oncolytic-deficient genotype VII NDV's 450th phenylalanine is mutated to leucine, its infectivity in tumor cells falls short of the naturally oncolytic genotype IV Herts/33. The mutated genotype VII NDV also exhibits slightly lower infectivity in non-tumor normal cells compared to naturally Herts/33 strain, emphasizing NDV's selective infectivity for tumor cells over normal ones. Importantly, the 450th amino acid does not impact NDV's infectivity in avian cells, aligning with existing research. Our work is dedicated to understanding NDV's oncolytic mechanisms without introducing risks to its host range or transmissibility. All experiments involving NDV were executed in the animal biosafety level 3 facility (CNAS registration No. CNAS BL0015) at Yangzhou University in strict accordance with the recommendations of the institutional biosafety manual** **and supervised by the Institutional Biosafety Committee of Yangzhou University. We are thankful for your recommendations, which have significantly enhanced our subsequent writing and submission. I wish that these explanations will clarify any concerns related to DURC. Please feel free to let me know if you have any further concerns regarding this matter.**

**We included information regarding biosafety and regulatory authority in lines 114-120 of the original text. We highly appreciate the opportunity to submit the revised manuscript. Thank you very much for your guidance and valuable recommendations.**

**Sincerely,**

**Prof. Dr. Xiufan Liu**

**Yangzhou University, Yangzhou, China**

**E-mail: xfliu@yzu.edu.cn**
